# Electrochemical biosensor for detection of 17β-estradiol using semi-conducting polymer and horseradish peroxidase

**DOI:** 10.1039/c9ra09902f

**Published:** 2020-03-03

**Authors:** Kamila Spychalska, Dorota Zając, Joanna Cabaj

**Affiliations:** Faculty of Chemistry, Wrocław University of Science and Technology Wybrzeże Wyspiańskiego 27 Wrocław Poland joanna.cabaj@pwr.edu.pl

## Abstract

A convenient electrochemical sensing pathway for 17β-estradiol detection was investigated. The system is based on a conducting polymer and horseradish peroxidase (HRP) modified platinum (Pt) electrode. The miniature estradiol biosensor was developed and constructed through the immobilization of HRP in an electroactive surface of the electrode covered with electroconducting polymer – poly(4,7-bis(5-(3,4-ethylenedioxythiophene)thiophen-2-yl)benzothiadiazole). The detection strategy is based on the fact that 17β-estradiol (E2) and pyrocatechol (H_2_Q) are co-substrates for the HRP enzyme. HRP, which does not react with E2, in the presence of H_2_O_2_ catalyses the oxidation of H_2_Q to *o*-benzoquinone (Q). With the optimized conditions, such constructed biosensing system demonstrated a convenient level of sensitivity, selectivity in a broad linear range – 0.1 to 200 μM with a detection limit of 105 nM. Furthermore, the method was successfully applied for hormone detection in the presence of potential interfering compounds (ascorbic acid, estriol, estrone, uric acid and cholesterol).

## Abstract 2

A convenient electrochemical sensing pathway for 17β-estradiol detection was investigated.

## Graphical abstract

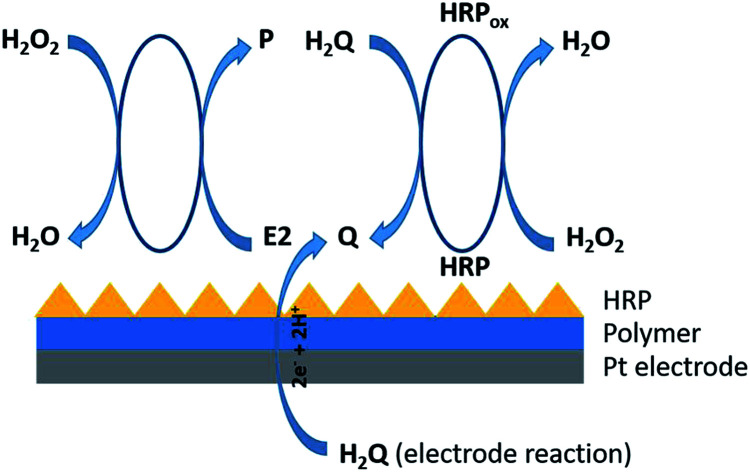

## Introduction

Many studies conducted in recent decades have shown that a lot of chemical substances – both those of natural origin as well as man-made ones – can interfere with the proper functioning of the endocrine system and cause health- and life-threatening effects on animals and humans. These compounds have been referred to as endocrine disrupting compounds (EDCs).^1^ These compounds can be found in many commercial products, such as food, medicines, cosmetics, detergents, food metal cans, toys, plastic bottles, pesticides and many others.^2^

**Fig. 1:**
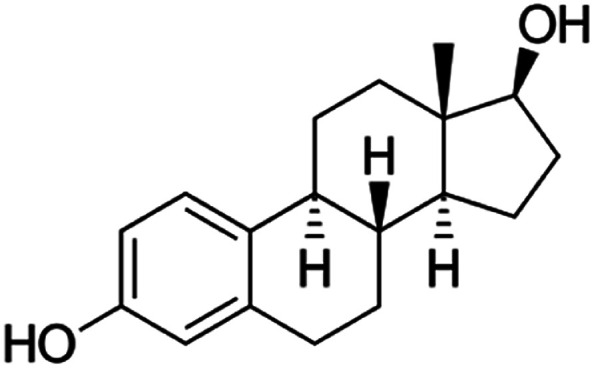
Chemical structure of 17β-estradiol.

17β-Estradiol (E2) (Fig. 1) and other naturally occurring hormones, natural chemicals, man-made chemicals and pharmaceutical products are classified as endocrine active compounds due to their ability to mimic endogenous hormones.^3^ Due to their unfavourable effect, they can interfere with the hormonal, immune and nervous systems of mammals.^4^ The concentration of EDC in the environment is small, however, chronic low exposure can cause harmful biological effects on animals and humans.^5^ To solve these problems, it is necessary to use simple, fast, sensitive and accurate methods for the determination of these combinations.

Horseradish peroxidase (HRP) is one of the most widely studied and most frequently used enzymes in the construction of enzyme biosensor systems, due to its easy availability and low cost. This enzyme contains heme as a prosthetic group, which is thus the active site of proteins with a resting state of iron (Fe) ion. HRP catalyzes the oxidation of many substrates, however, it is very often used for the oxidation of phenolic compounds.^6^ HRP belongs to the class of oxidoreductases in which the electron acceptor is hydrogen peroxide. This enzyme is used on a large scale due to commercial availability in high purity, which allows to study biological behaviors that catalyze the oxidation of substrates in the presence of H_2_O_2_. It is used, for example, in the production of amperometric sensors detecting H_2_O_2_ and small organic and inorganic substrates.^7^

An extremely important task in the production of amperometric sensors is the efficient and effective immobilization of the biofilm on the surface of the electrode. Conducting polymers deserve particular attention due to their redox, optical, mechanical and electrical properties. Due to their high durability and stability, they are a suitable material for the construction of sensor devices.^8,9^ The performance of the biosensor depends mainly on the structure of the surface, the interaction between the enzyme and the surface of the electrode and the protection of the three-dimensional structure of the protein. Therefore conductive polymers have proven to be one of the most useful transducers due to their simplicity of production. Conducting polymers act as a three-dimensional matrix for deposition of biomolecules.^10^

Different methods are used for hormone determination which are reported in the literature such as high-performance liquid chromatography (HPLC),^11^ capillary electrophoresis,^12^ UV spectrophotometry^13^ or gas chromatography combined with mass spectroscopy (GC-MS).^14^ However, commonly used techniques for determination of EDCs, such as HPLC or GC-MS are time-consuming, very expensive and often not accurate. Electrochemical biosensors have recently gained popularity because of a number of advantages such as low cost, fast response, ease of use and real-time analysis.^15^ Electrochemical biosensors for 17β-estradiol detection are widely described in the literature, however, only few items deal with electrochemical biosensors with the enzyme as a biologically active element.^16,17^ Wang *et al.* proposed an electrochemical biosensor for the detection of 17β-estradiol based on electropolymerized l-lysine molecules on a glassy carbon electrode (GCE) modified with citric acid and graphene (CA-GR) and cross-linked with laccase. This biosensing system could effectively determine the concentration of 17β-estradiol in tested sample with LOD 1.3 × 10^−13^ M.^18^ Another detection system based on laccase was proposed by Povedano *et al.* For this purpose they used glassy carbon electrode coated with nanocomposite based on graphene oxide with rhodium nanoparticles. On the surface of such prepared electrode, the enzyme laccase was successfully anchored to construct a voltammperometric biosensor for 17β-estradiol determination. Such constructed system was able to measure the concentration of 17β-estradiol in the 0.9–11 pM range with LOD of 0.54 pM.^19^

Herein, we present a simple and very sensitive electrochemical enzyme – based biosensor for determination of 17β-estradiol (E2). Horseradish peroxidase (HRP), due to the low cost and high availability, is one of the most extensively investigated enzymes in the case of the development of biosensors. HRP-based biosensors present the highest sensitivity for a number of phenol derivatives since they can act as electron donors for enzyme regeneration. The use of the enzyme in the system significantly reduces the time of analysis. In the case of biosensors in which antibodies are a biologically active element, very often before proceeding to the proper measurement, prior incubation with the analyte to form antibody–antigen complexes is required. The regeneration of the electrode after the measurement is also significant. In the case of enzymatic biosensors, measurements can be carried out continuously, very often only rinsing the electrode in a medium containing no analyte is required. In the case of immunosensors, it is necessary to use more radical reagents that will break the antibody–antigen binding while preserving the antibodies on the electrode surface. Each subsequent radical treatment of the electrode may cause detachment of antibodies from the electrode surface, thereby shortening the lifetime of the constructed system.

**Fig. 2:**
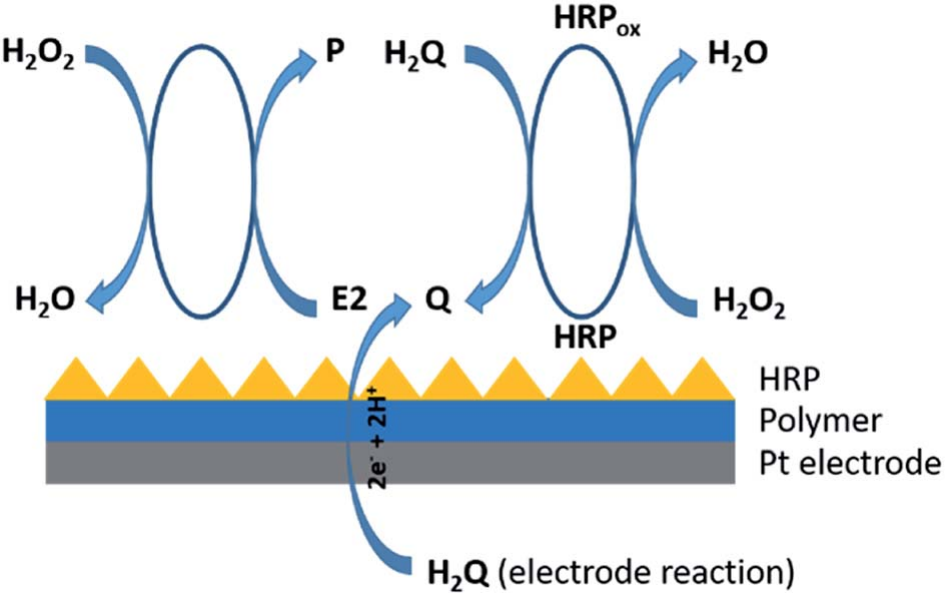
Schematic representation of the 17β-estradiol enzyme-based biosensor.

The sensor was constructed through the immobilization of the enzyme – horseradish peroxidase (HRP) on the surface of thin polymer layer based on poly(4,7-bis(5-bromothiophen-2-yl)benzothiadiazole). Such prepared layered electrode was transferred into an electrochemical cell containing pH 7.00 phosphate buffer, where given amounts of HRP, pyrocatechol (H_2_Q), H_2_O_2_ and 17β-estradiol (E2) were added. H_2_Q and E2 are both enzyme co-substrates. In the presence of H_2_O_2_, HRP catalyzes the oxidation of H_2_Q to benzoquinone (Q) and E2 to given product.^20^ The electrochemical response of the system is proportional to H_2_Q concentration and inversely proportional to the E2 concentration in the test samples. Therefore, the maximal electrochemical response was obtained with the minimum concentration of E2 in the sample analyzed (Fig. 2).

## Materials and methods

### Reagents and materials

Horseradish peroxidase (HRP) (EC 1.11.1.7), 17β-estradiol (E2), uric acid (UA), ascorbic acid (AA), estriol (E3), estrone (E1), cholesterol (CH), tetrabutylammonium hexafluorophosphate (TBA-HFP), *n*-butyllithium (2.5 M in hexane), bis(triphenylphosphine)palladium(ii) dichloride (99%), 3,4-ethylenedioxythiophene (97%) as well as 4,7-bis(5-bromothiophen-2-yl)benzothiadiazole (99%) were purchased from Sigma-Aldrich Co. Dichloromethane, KH_2_PO_4_, NaH_2_PO_4_, ethanol and anhydrous tetrahydrofuran were purchased from POCH. Monomer 4,7-bis(5-(3,4-ethylenedioxythiophene)thiophen-2-yl)benzothiadiazole was synthesized according to the method described below. All buffers, which were needed to wash an unbounded protein and to prepare the measurement solution were prepared according to generally known, obligatory standards. All chemicals were analytically pure and used without any further purification. Preparative column chromatography was performed on the glass column with Acros Organics silica gel for chromatography, 0.035–0.075 mm 60 Å. 600 MHz ^1^H NMR and ^13^C NMR spectra were recorded in deuterated chloroform (CDCl_3_) on Bruker Avance II 600 Instruments, respectively. Chemical shifts were locked to chloroform *δ*_H_ 7.26 (s) and *δ*_C_ 77.16 (t) signals. The molecular weight of the final product was determined using a Bruker micrOTOF-Q spectrometer, FWHM-17500, 20 Hz. The 2-(tributylstannyl)-3,4-ethylenedioxythiophene (2) were synthesized according to previously published procedures.^17^

### Apparatus and electrodes

The platinum electrode (Pt, diameter 3 mm, produced by BASi) was polished before the experiment with 3 μm fine diamond polish and rinsed thoroughly with double distilled water. Then, it was immersed in a solution of H_2_SO_4_ + H_2_O_2_ (3 : 1 v/v) during 5 min. Finally, it was rinsed with water and ethanol, and air dried. The counter electrode (CE) was a platinum wire. Ag/AgCl electrode saturated in 4 M KCl was used as a reference electrode. The measuring system for performing DPV, CV and chronoamperometry was Autolab PGSTAT 128 potentiostat run with the GPES software. All DPV and CV measurements were performed in the potential range from −0.2 to 0.7 V (step potential 0.00495 V, modulation amplitude: 0.04995 V).

### Synthesis and characterization of monomer 4,7-bis(5-(3,4-ethylenedioxythiophene)thiophen-2-yl)benzothiadiazole

To a mixture of 4,7-bis(5-bromothiophen-2-yl)benzothiadiazole (1) (1.00 g, 2.18 mmol) and 2-(tributylstannyl)-3,4-ethylenedioxythiophene (2) (2.07 g, 4.80 mmol) in anhydrous THF (80 mL) was added bis(triphenylphosphine)palladium(ii) dichloride (Pd(PPh_3_)_2_Cl_2_) (0.306 g, 0.436 mmol) at room temperature under nitrogen atmosphere. The resulting mixture was refluxed with stirring for 48 h. Then the reaction mixture was concentrated under reduced pressure, diluted with water, and extracted with EtOAc. The extract was washed with brine, dried over MgSO_4_, and concentrated. The residue was purified by silica gel column chromatography (hexane–EtOAc) to give 3 (1.075 g, 84.6%) as a purple solid state.

Mp: 143–145 °C.

^1^H NMR (400 MHz, CDCl_3_), *δ* (ppm): *δ* 8.07 (d, *J* = 4.0 Hz, 2H), 7.83 (s, 2H), 7.30 (d, *J* = 4.0 Hz, 2H), 6.27 (s, 2H), 4.41–4.39 (m, 4H), 4.28–4.26 (m, 4H).

^13^C NMR (151 MHz, CDCl_3_), *δ* (ppm): *δ* 150.15, 146.93, 132.46, 132.24, 130.89, 130.52, 128.80, 128.48, 123.76, 114.70, 101.66, 90.06, 68.72.

MS (*m*/*z*): [M^+^] 580.9799.

### Modification of electrode

The Pt electrode was modified with a thin layer of poly(4,7-bis(5-(3,4-ethylenedioxythiophene)thiophen-2-yl)benzothiadiazole) and HRP. The electropolymerization process of monomer was carried out using a potentiostat/galvanostat AUTOLAB PGSTAT128N with GPES software in a typical three-electrode electrochemical cell (10 mL) appointed with a working platinum electrode, Ag/AgCl reference electrode (saturated in 4 M KCl), and a coiled platinum wire as the counter electrode. In order to synthesize the polymeric layer onto the surface of the clean electrode, 4,7-bis(5-(3,4-ethylenedioxythiophene)thiophen-2-yl)benzothiadiazole (1 mM) was dissolved in a dichloromethane solution containing 0.1 M tetrabutylammonium tetrafluoroborate (TBA-TFB). The electrodes were dipped in 8 mL of the monomer solution. The polymer film electrodeposition was performed using chronoamperometry method (potential 1.5 V, duration 600 s). Then, the polymer layer was washed gently with dichloromethane and pH 7.00 phosphate buffer.

In the next step, HRP was immobilized on the surface of the modified electrode by physical adsorption and covalent cross-linking with glutaraldehyde. HRP solution (3.0 mg mL^−1^) was prepared in a pH 7.00 phosphate buffer. The immobilization process was carried out for 24 hours, by the immersion of electrode in glutaraldehyde solution (2%) for 1.5 h. Then electrode was immersed in the solution of enzyme for 22 h. The unbound protein was washed by repeatedly plunging the electrode in pH 7.00 phosphate buffer. The prepared enzyme-modified electrode was stored in a phosphate buffer at an optimal pH at 4 °C.

### Optimal enzyme working conditions

In order to determine the optimal pH of enzyme work, a number of buffers were prepared – acetate with a pH of 4.0 and 5.0 and phosphate with a pH of 6.0, 7.0 and 8.0. Catalytic activity was measured by spectrophotometry method. The substrate for the enzyme was a solution of *ortho*-phenylenediamine in acetate or phosphate buffer and hydrogen peroxide. The reaction was carried out on a magnetic stirrer for 15 minutes for each buffer at 470 nm, taking 1 mL of absorbance measurement solution every 30 seconds for the first 5 minutes of the reaction and then every 1 minute.

### Electrochemical measurements

17β-Estradiol detection was performed using CV and DPV method with a potentiostat/galvanostat AUTOLAB PGSTAT 128N with GPES software. The measurements were conducted with typical three-electrode system in 10 mL cell. Platinum electrode (modified with monomer and enzyme) was used as working electrode, together with a coiled platinum wire as the auxiliary electrode and an Ag/AgCl reference electrode. CV and DPV were carried out by repeated potential scanning in range −0.2 to 0.7 V with scan rate: 50 mV s^−1^ in the presence of different concentration of 17β-estradiol dissolved in 0.1 M phosphate buffer pH = 7.0 (0.1–200 μM). All electrochemical measurement were carried out in room temperature and air-opened conditions.

## Results

### Synthesis and characterization of monomer 4,7-bis(5-(3,4-ethylenedioxythiophene)thiophen-2-yl)benzothiadiazole

**Scheme 1:**
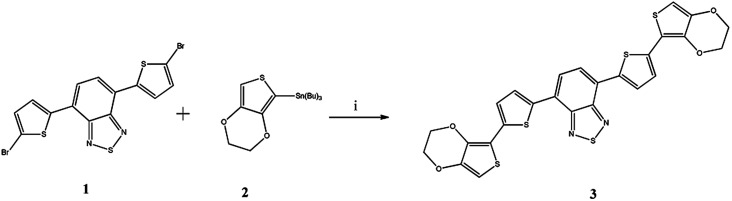
The synthetic route of the 4,7-bis(5-(3,4-ethylenedioxythiophene)thiophen-2-yl)benzothiadiazole (3). (i) Pd(PPh_3_)_2_Cl_2_, THF, N_2_, 48 h.

The detail of synthetic route for new benzothiadiazole derivative is shown in Scheme 1. The designed macrostructure 3 was synthesized by palladium catalyzed Stille coupling reaction of 4,7-bis(5-bromothiophen-2-yl)benzothiadiazole (1) and 2-(tributylstannyl)-3,4-ethylenedioxythiophene (2) with excellent yield (85%).

Conducting polymers (CPs) are an interesting alternative to creating matrices in biosensor systems. Due to the presence of conjugated π electrons (a system having coupled C=C bonds) CP are characterized by unique electronic properties, such as: high electron affinity, low optical transition energy or low ionization potential. Importantly, CPs serve as electron mediators to improve the flow of electrons between the enzyme's active center and the electrode surface. They create an appropriate microenvironment to immobilize the protein and act as a transducer during the transfer of electric charge.^9,21^ In fact, CP is easy to manipulation of their electrical and physicochemical characteristics through reduction or oxidation process (n-type or p-type materials).^22^ Furthermore, conductive polymers have a hydrophobic backbone that facilitates stackable π–π molecules, making the interaction between the protein and the polymer matrix stronger. The use of conducting polymers as matrix molecules allows to increase the stability, speed and sensitivity of constructed sensor devices.^23^ In addition to electronic properties, CPs are also characterized by low synthesis price and universality – their properties can be modified by physical changes (such as pH or temperature), even after the synthesis process.^24,25^ Moreover, we can investigate the effects of modifications in chemical structures of CP. Furthermore, one of the most attractive p-type semiconductors, 3,4-ethylenedioxythiophene (EDOT) has been demonstrated as a neutral electrode or electroactive scaffold for protein^26^ and have advanced stability due to the exclusion of oxidative-damage-based inactivation.^27^

**Fig. 3:**
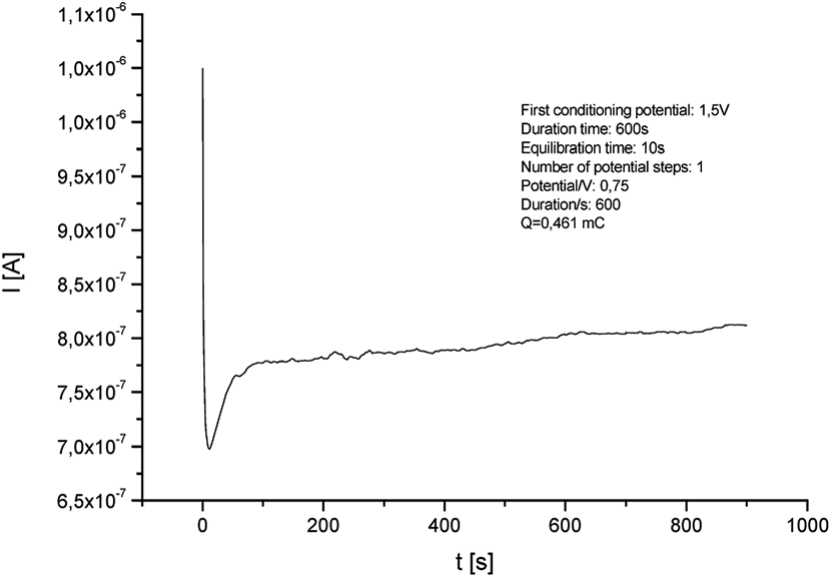
Chronoamperogram of electrochemical deposition of poly[4,7-bis(5-(3,4-ethylenedioxythiophene)thiophen-2-yl)benzothiadiazole] on the surface of Pt electrode (potential: 1.5 V, duration: 600 s).

Electrodeposition of the monomer was provided with the chronoamperometric method (Fig. 3), which allows to obtain controlled thin layer of polymer film on the surface of the platinum electrode. The Ag/AgCl electrode was used as a reference electrode. Surface modification with enzyme and platform for anchoring the protein is a key step during the construction of biosensors. The enzymatic activity depends on the chemical species used, which are a matrix for the immobilization of biocatalyst. During the process, an increase in current values is visible during the passage of time. This demonstrates an effective electropolymerization of 4,7-bis(5-(3,4-ethylenedioxythiophene)thiophen-2-yl)benzothiadiazole – a p-type polymer. As a result, a thin layer of 50 nm polymer film was obtained on the surface of Pt electrode. The thickness of the layer was calculated using the eqn (1):1
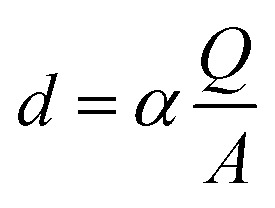
where: *d* – thickness of the polymer layer [nm], *α* – constant characteristic for a given polymer [cm^2^ nm mC^−1^] (for polythiophene-based polymers *α* = 2,5), *Q* – charge [mC], *A* – electrode surface [cm^2^].

**Fig. 4:**
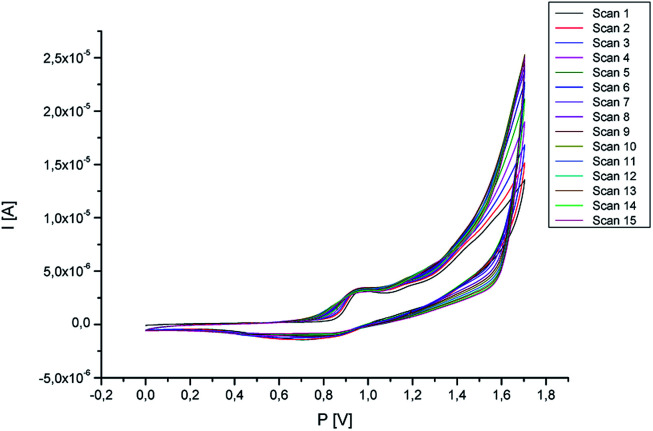
Stability of the polymer (potential range: 0–1.6 V, scan rate 50 mV s^−1^, number of scans: 10, supporting electrolyte: TBA-TFP solution in dichloromethane).

The study of the stability of the polymer covering the surface of the working electrode with cyclic voltammetry was also carried out. For this purpose, 15 measurement cycles were carried out in the potential range 0–1.7 V, as the scanning speed was set at 50 mV s^−1^. The test was carried out in a 0.1 M TBA-TFP solution in dichloromethane. Along with the subsequent measurement cycles, no significant changes in the current intensity were observed (Fig. 4). This confirms the stability of the formed polymer.

**Fig. 5:**
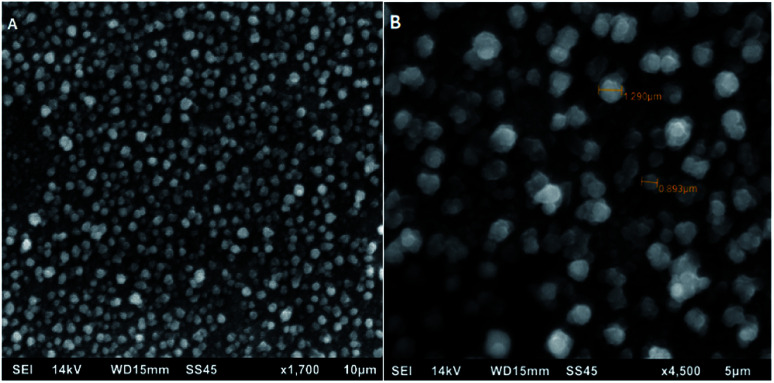
SEM images for poly[4,7-bis(5-(3,4-ethylenedioxythiophene)thiophen-2-yl)benzothiadiazole] 10 μm (A) and 5 μm (B).

The electropolymerization process and effective deposition of poly[4,7-bis(5-(3,4-ethylenedioxythiophene)thiophen-2-yl)benzothiadiazole] on the surface of Pt electrode was also confirmed using scanning electron micrographs (SEM) displayed in Fig. 5. Micrographs confirm the formation of a polymer with unobservable surface defects and a granular structure characteristic of benzothiadiazole derivatives. The diameter of the resulting grains is about 1 μm. Such a pore diameter is able to allow a much smaller enzyme molecule (resolution 1.57 Å to 0.000157 μm) to anchor freely in this structure while maintaining its catalytic activity due to the lack of formation of covalent bonds and possible rotation to direct the active site of the enzyme towards the substrate. The above information confirms that semiconducting polymers can be successfully used as a basic element in the construction of biosensor devices – not only due to semi-conductive properties – but also as a matrix for anchoring the enzyme.

### Electrochemical measurements

**Fig. 6:**
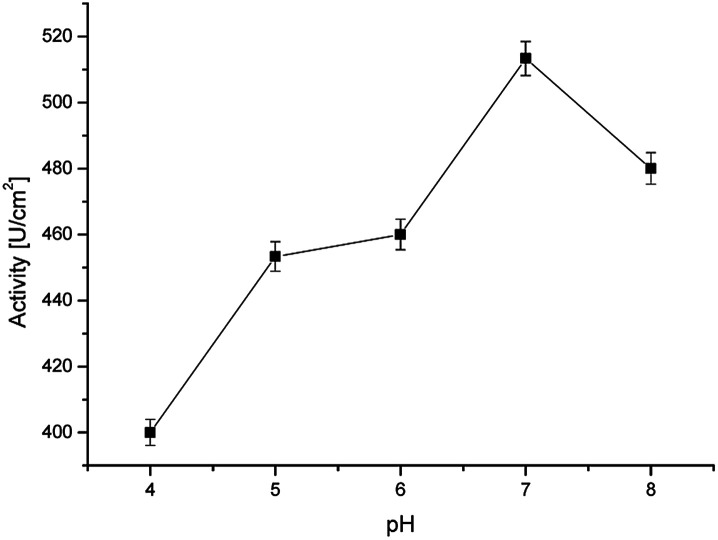
Activity of the immobilized horseradish peroxidase at various pH values (at room temperature).

For the measurements, 17β-estradiol was dissolved in ethanol and then in pH 7.00 phosphate buffer for two reasons. First, the buffer creates an optimal environment for real sample testing, and secondly – the buffer is the most optimal for horseradish peroxidase activity. As shown in Fig. 6, optimal conditions for horseradish peroxidase activity is pH 7.0, where the enzyme activity is the highest at 60 °C.^28^ However, due to the fact that the sensor is intended to be used in point of care diagnostics, where it is often impossible to raise the temperature to 70 °C, it was decided that the solutions will be prepared in the given buffer and the measurements will be carried out at room temperature.

**Fig. 7:**
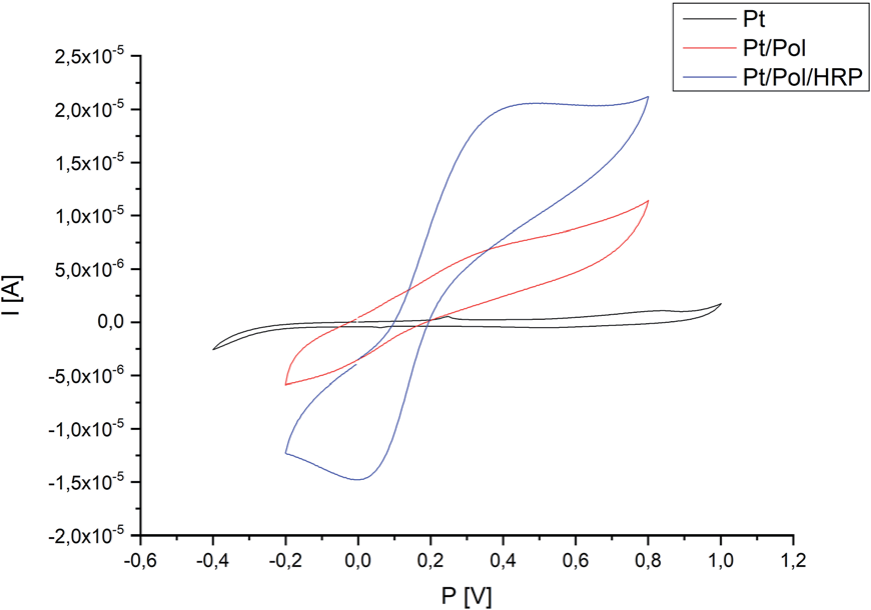
Representative CV-scans of the bare Pt electrode, Pt electrode modified with polymer and Pt/Pol/HRP system in the presence of 17β-estradiol 10(μM) under applied potential range (potential range −0.4 to 1.0 V, scan rate 100 mV s^−1^, *via* Ag/AgCl).

Fig. 7 presented the cyclic voltammograms (CVs) of different electrodes: a bare platinum electrode (Pt), a poly(4,7-bis(5-(3,4-ethylenedioxythiophene)thiophen-2-yl)benzothiadiazole) (Pol) modified Pt electrode and the Pt/Pol/HRP complex, recorded in the presence of a 10 μM solution of 17β-estradiol. As shown in Fig. 7, evident electrochemical redox peak of the bare Pt electrode and Pt/Pol in the PBS solution couldn't be observed. While, a reduction peak at about 0.05 V and an oxidation peak at 0.4C appeared at the Pt/Pol/HRP electrode. The HRP is a kind of common electron transfer protein, and HRP immobilized on the surface of poly[4,7-bis(5-(3,4-ethylenedioxythiophene)thiophen-2-yl)benzothiadiazole] in a certain extend can promote electron transfer between semi-conductive polymer and the electrode. The good electron transport material based on poly[4,7-bis(5-(3,4-ethylenedioxythiophene)thiophen-2-yl)benzothiadiazole] retrieved a very important role in promoting direct electrochemistry of HRP. At the same time, the synthesized material is a good support material and can load a large number of HRP molecules and keep its activity, so the electrosynthesized polymer influences positively on the electronic transfer of HRP.

The detection basis in the constructed system is the Q reduction reaction to H_2_Q according to the reaction equation: Q + 2e + 2H^+^ → H_2_Q. 17β-estradiol and H_2_Q are a phenolic compounds with a hydroxyl group located at carbon 3 and both are co-substrates for HRP. Research conducted by Molina *et al.* confirmed that HRP is able to recognize 17β-estradiol as a co-substrate in a homogenous system. In this article, higher net peak currents were observed at lower E2 concentrations, indicating that HRP catalyzes the oxidation of H_2_Q to Q.^20^ As E2 concentration increases, a decrease in peak current is observed, confirming that HRP reacts with both H_2_Q as well as with E2. The current values corresponding to the enzymatically produced Q are inversely proportional to the amount of E2 in test samples. Therefore, the maximal electrochemical response was obtained in the absence of 17β-estradiol in the tested sample.

### Optimization of the concentrations of species involved in the reaction of the biosensor

**Fig. 8:**
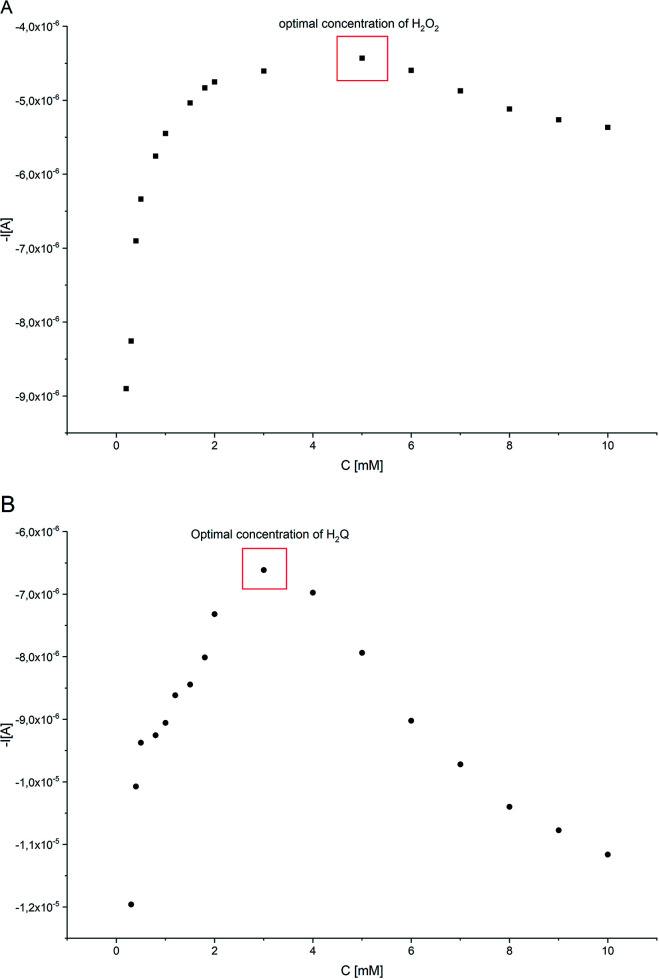
Variation of the *I*_p,n_ for 100 μM of E2 solution in constructed system as a function of: (A) C*H_2_O_2_ (C*H_2_Q = 1.0 × 10^−3^ M, C*HRP = 1.5 × 10^−10^ M); (B) C*H_2_Q (C*H_2_O_2_ = 1.0 × 10^−3^ M, C*HRP = 1.5 × 10^−10^ M).

Fig. 8A shows the effect of H_2_O_2_ concentration change at a given concentration of H_2_Q (1.0 × 10^−3^ M), HRP (1.5 × 10^−10^ M) and concentration of 17β-estradiol 1 × 10^−4^ M. The optimal H_2_O_2_ concentration was found as 5 × 10^−3^ M. The effect of H_2_Q concentration at given concentrations of HRP, H_2_O_2_ and 17β-estradiol was also analyzed (Fig. 8B). The optimal H_2_Q concentration was 3 × 10^−3^ M for HRP (1.5 × 10^−10^ M), H_2_O_2_ (5 × 10^−3^ M) and 17β-estradiol (1 × 10^−4^ M), respectively. These optimal H_2_O_2_ and H_2_Q amounts were used for further experiments.

### Calibration curve for 17β-estradiol. Analytical performance

**Fig. 9:**
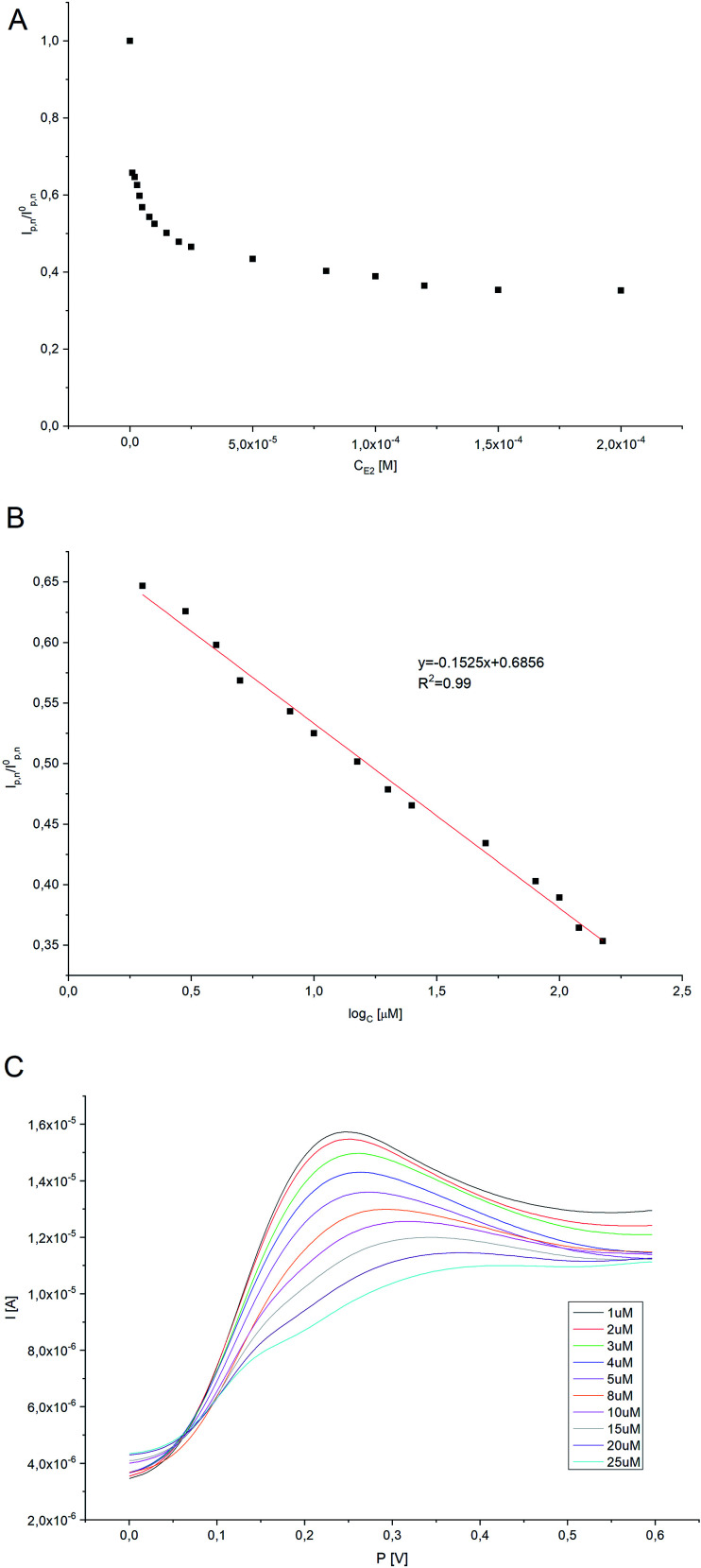
(A) The curve of *I*_p,n_/*I*_0p,n_ corresponding to the concentrations of E2 (where *I*_p,n_ is the net peak current in the presence of E2, *I*_0p,n_ is the net peak current in the absence of E2). (B) The linear relationship between *I*_p,n_/*I*_0p,n_ and the logarithm of E2 concentration. (C) Typical DPV responses for E2 with different concentrations (from a to j: 1 μM, 2 μM, 3 μM, 4 μM, 5 μM, 8 μM, 10 μM, 15 μM, 20 μM, 25 μM, respectively).

Electrochemical character of 17β-estradiol was investigated employing DPV method (potential range −0.2 to 0.8 V) in oxygen-saturated conditions. The higher net peak currents were observed at lower E2 concentrations, indicating that HRP catalyzes the oxidation of H_2_Q to Q. As E2 concentration increases, a decrease in peak current is observed, confirming that HRP reacts with both H_2_Q as well as with E2. The current values corresponding to the enzymatically produced Q are inversely proportional to the amount of E2. As the concentration of estradiol increases, the H_2_Q oxidation peak practically disappears on the modified electrode, which was the expected effect. A titration curve was performed for various concentrations of 17β-estradiol (0.1–200 μM) (Fig. 9A). The amperometric response for the 17β-estradiol detection showed to be effective in the range form 0.1–200 μM and a linear calibration curve was obtained by plotting the ratio of *I*_p_ (in the presence of estradiol)/*I*_p0_ (in the absence of estradiol) to the logarithm of the 17β-estradiol concentration added. The linear interval can be expressed by the equation *y* [*I*/*A*] = −0.1525[log[E2]] + 0.6858 (Fig. 9B and C). Limit of detection for constructed survey (LOD) has been calculated as (2):^23^

2 LOD = 3.29*σ*_B_/*b*

where: *σ*_B_ is the standard deviation of the population of blank response, *b* is the slope of the regression line. In this way LOD was calculated and found to be 105 nM. In Table 1, the characteristic of proposed electrode is highlighted with those presented in the literature for estrogens determination.

**Table 1:** Reported electrodes for estrogens determination

Electrode	Biological component	Method	LOD	Linear range [μM]	Sensitivity	Recovery	Ref.
Fe_3_O_4_@Au-GSH/MIPs/GCE	×	DPV	2.76 × 10^−3^ μM	0.025–10	0.58 μA μM^−1^	>85%	29
MIPs/PtNPs/GCE	×	DPV	1.6 × 10^−2^ μM	0.03–50	0.79 μA μM^−1^	>90%	30
MIPs/PtNPs/rGO/GCE	×	DPV	2.0 × 10^−3^ μM	0.04–50	×	>90%	31
CuThP/rGO/GCE	×	DPV	5.3 × 10^−3^ μM	0.1–1	0.39 μA μM^−1^	>95%	32
BPIDS/GCE	×	DPV	5.0 × 10^−2^ μM	0.1–10	0.19 μA μM^−1^	>95%	33
AuNPs/ceramic biosensor	ER-α	CV	2.6 × 10^−2^ μM	0.001–0.032	×	>90%	34
CuO/CPE	×	SWV	2.1 × 10^−2^ μM	0.06–0.8	3.409 μA M^−1^	>90%	35
GNR-FS-Au-CA/CPE	E2-specific aptamers	DPV	7.4 × 10^−3^ μM	0.1–5	×	>90%	36
rGO-DHP/GCE	×	CV	7.0 × 10^−2^ μM	0.4–5.0	1.65 μA M^−1^	>95%	37
Pt/Pol/HRP	HRP	DPV	105 nM	0.1–200	1.16 × 10^−4^ A μM^−1^cm^−2^	>90%	This work

Compared with the detection methods described in Table 1, the enzyme-based biosensor presented has good sensitivity and a relatively wide linear range. The detection limit of the current sensor is good enough and has many advantages such as low-cost materials – all reagents necessary for the construction of the biosensor are rather cheap and easily available compared to systems using a larger number of biologically active elements (*e.g.* systems containing antibodies or receptors), relatively easy synthesis of semi-conductive material – properly optimized method allows obtaining material with excellent yield (around 85%) in a short time, and easy manufacturing. Moreover, the use of the enzyme in the constructed system significantly reduces the time of analysis by skipping often time-consuming steps, such as the incubation of the electrode with the target analyte (in the case of immunosensors and systems based on receptors) or regeneration of the electrode to free places of interaction with the analyte before proceeding to the next measurement. Such created system is characterized by high stability of both the polymer matrix covering the electrode surface as well as the enzymatic protein immobilized on its surface and repeatability of results.

The limit of quantification (LOQ) was also determined using eqn (3) and it equalled 159.57 nM.

3 LOQ = 5*σ*_B_/*b*

In which *σ*_B_ is the standard deviation of the population of blank response, *b* is the slope of the regression line.^23^ Furthermore, sensitivity of the proposed biosensor was found to be 1.16 × 10^−4^ A μM^−1^ cm^−2^. All parameters demonstrating an analytical validation are shown in Table 2.

**Table 2:** Analytical performance

Linearity	LOD	LOQ	*R* ^2^	Slope	SD of slope	Intercept	SD of intercept	Sensitivity
0.1–200 μM	105 nM	159.57 nM	0.99	3.65 × 10^−6^	8.52 × 10^−8^	1.65 × 10^−5^	1.20 × 10^−7^	1.16 × 10^−4^

The increase in 17β-estradiol concentration in the measurement system led to saturation (plateau) of the system on the calibration plot. Because of this, Michaelis–Menten kinetics analysis was also performed. The apparent Michaelis–Menten *K*_m_ constant characterizing the enzyme-analytics kinetics was determined using the Lineweaver–Burk equation (eqn (4)), described as:4
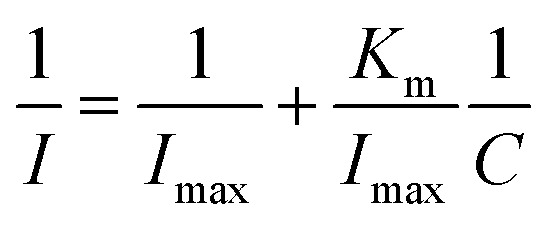
where *I* is the steady-state current reached after addition of the analyte (substrate), *I*_max_ is the maximum current measured under saturated conditions, and *C* is the analyte concentration. The *K*_m_ constant was found to be 6.83 × 10^−5^ M. The *K*_m_ constant indicates that horseradish peroxidase immobilized onto the Pt/Pol platform retains its bioactivity towards 17β-estradiol.

**Fig. 10:**
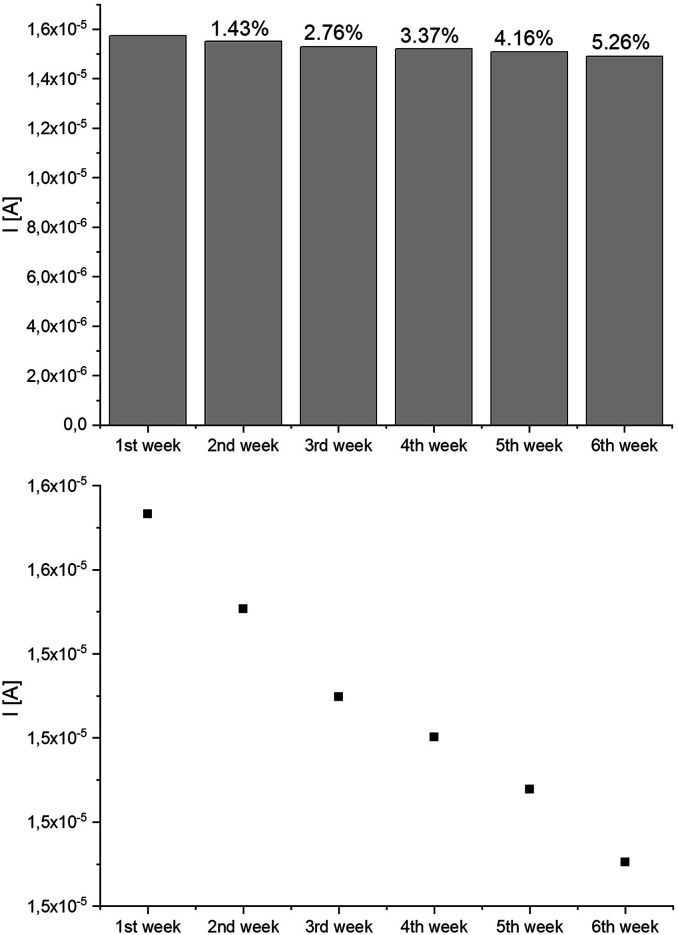
Lifetime analysis of constructed system.

The analysis of the lifetime of the constructed system was also carried out. The lifetime of enzymatic biosensors is limited by the loss of enzyme activity over time. For this purpose, the system response to 1uM E2 concentration was measured at 1 week intervals. The system was considered stable until the change in peak current did not exceed 5%. As can be deduced from the graph presented in Fig. 10 the system lost its activity over time, which at the turn of the 5th and 6th week exceeded 5%. Therefore, the lifetime of the system can be determined for 5 weeks.

### Determination of 17β-estradiol in the presence of interfering substances

The selectivity and specificity of a biosensor are very important parameters in the context of correct design bio-devices. It means, that the biosensor should not be subjected to interference by other hormones or biomarkers while in use. In this project, we were studied five different substances (ascorbic acid (AA), estriol (E3), estrone (E1), uric acid (UA) and cholesterol (CH)) as a potential interference compounds during the detection of 17β-estradiol. The justification of selecting previously mentioned substances in this investigation was based on the similar chemical structure between 17β-estradiol and potential interference substances, and also the presence of such compounds in the human body was taken into account. All presented specimens were added in a concentration equalled to 50 μM to different E2 samples (10, 50 and 100 μM) to check the influence of these compounds in large excess, equilibrium and deficiency of interfering substances. Each tested reagent has an insignificant effect (<10%) on the peak current of the samples compared to the blank (0% AA, 6% E3, 9% E1, 5% UA and 2% CH).

**Fig. 11:**
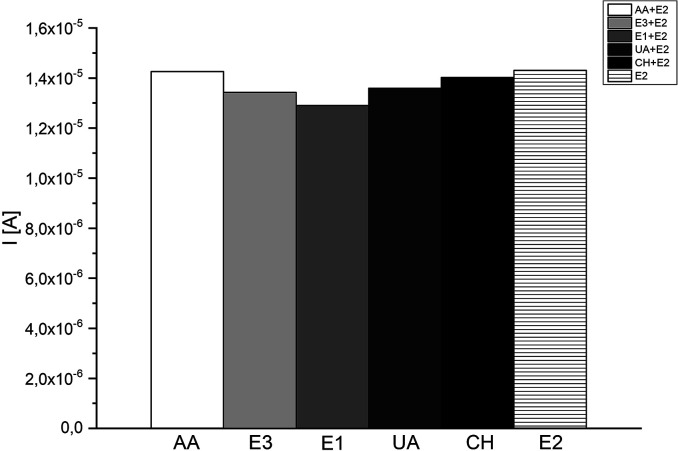
Effect of interfering substances (50 μM) on the determination of E2 level.

Presented results (Fig. 11) affirm insignificant impact on the selectivity of constructed system, and convict that checking interferences does not disturb the work of proposed 17β-estradiol test. Furthermore, presence of HRP in the detection system, allows the efficient examination of lower 17β-estradiol concentrations (>10 μM) and interfering species have insignificant effect on the measurements, because of the enzymes selective nature. As a result, the biosensor exhibited adequate selectivity for 17β-estradiol determination.

### Pharmaceutical sample analysis

To evaluate the practicability of the presented procedure, electrochemical analysis was used to determine the estradiol level in the labelled pharmacological product (*Estradiolum*, 2 mg, Novo Nordisk A/S). The analytical results for testing samples are given in Table 3. A very promising recovery value (ratio of the determined concentration to the actual concentration of 17β-estradiol in the sample, expressed in %), clearly confirms the efficiency of the proposed method for the useful detection of 17β-estradiol.

**Table 3:** Detection effect of 17β-estradiol based on the method presented

Real sample	*C*_detected_	*C*_calculated_	Recovery (%)	RSD
*Estradiolum*, 2 mg, Novo Nordisk A/S	0.97 μM	1 μM	102.69	±2.43%
	25.94 μM	25 μM	96.37	
	53.69 μM	50 μM	93.12	

## Conclusions

The study showed a method for producing an electrochemical horseradish peroxidase biosensor for the detection of 17β-estradiol. The platinum electrode was modified with a previously synthesized conducting polymer, which was the appropriate matrix for immobilization of horseradish peroxidase. The enzyme was attached to the substrate and retained high catalytic activity. Electrochemical measurements have shown that the designed system works with high accuracy and reliability. The detection limit for 17β-estradiol was set to 105 nM. No interference was found with AA, E3, E1, UA, CH. The main advantages of the presented sensor are: sensitivity, precision, linearity and simplicity of the structure.

## Conflicts of interest

There are no conflicts to declare.

## Supplementary Material

## References

[cit1] La Spina R., Ferrero V. E. V., Aiello V., Pedotti M., Varani L., Lettieri T., Calzolai L., Haasnoot W., Colpo P. (2018). Biosensors.

[cit2] Kortenkamp A., Martin O., Faust M., Evans R., McKinlay R. F., Orton F., Rosivatz E. (2011). Final Rep..

[cit3] Waring R. H., Harris R. M. (2005). Mol. Cell. Endocrinol..

[cit4] Jeselsohn R., Yelensky R., Buchwalter G., Frampton G., Meric-Bernstam F., Gonzalez-Angulo A. M., Ferrer-Lozano J., Perez-Fidalgo J. A., Cristofanilli M., Gomez H. (2014). Clin. Cancer Res..

[cit5] Gomes R. L. and Lester J. N., Endocrine disrupters in drinking water and water reuse, CRC Press, London, UK, 2002, p. 219

[cit6] Joseph S., Rusling J. F., Lvov Y. M., Friedberg J., Fuhr Y. M. (2000). J. Anal. Chem..

[cit7] Hu S. , Lu Q. and Xu Y., Electrochemical Sensors, Biosensors and their Biomedical Applications, 2008, p. 531

[cit8] Ucan D., Kanik F. E., Karatas Y., Toppare L. (2014). Sens. Actuators, B.

[cit9] Baluta S., Malecha K., Zając D., Sołoducho J., Cabaj J. (2017). Sens. Actuators, B.

[cit10] Gokoglan T. C., Soylemez S., Kesik M., Toksabay S., Toppare L. (2015). Food Chem..

[cit11] Regan F., Moran A., Fogarty B., Dempsey E. (2003). J. Chromatogr. A.

[cit12] Yilmaz B., Kadioglu Y. (2013). Arabian J. Chem..

[cit13] Ming W., Wang H., Lu W., Zhang Z., Song X., Li J., Chen L. (2017). Sens. Actuators, B.

[cit14] Dai Y., Liu C. (2017). Biosensors.

[cit15] Dwiecki K., Nogala-Kałucka M., Polewski K. (2013). Żywność. Nauka. Technologia. Jakość.

[cit16] Fernstrom J. D., Fernstrom M. H. (2007). J. Nutr..

[cit17] Pander M. P., Data P., Turczyn R., Lapkowski M., Swist A., Soloducho J., Monkman A. P. (2016). Electrochim. Acta.

[cit18] Wang A., Ding Y., Li L., Duan D., Mei Q., Zhuang Q., Cui S., He X. (2019). Talanta.

[cit19] Povedano E., Cincotto F. H., Parrado C., Diez P., Sanchez A., Canevari T. C., Machado S. A. S., Pingarron J. M., Villalonga R. (2017). Biosens. Bioelectron..

[cit20] Monerris M. J., Arévalo F. J., Fernández H., Zon M. A., Molina P. G. (2015). Sens. Actuators, B.

[cit21] Nazari M., Kashanian S., Rafipour R. (2015). Spectrochim.
Acta, Part A.

[cit22] Schultze J. W., Karabulut H. (2004). Electrochim. Acta.

[cit23] Desimoni E., Brunetti B. (2013). Electroanalysis.

[cit24] Kolpin D. W., Furlong E. T., Meyer M. T., Thurman E. M., Zaugg S. D., Barber L. B., Buxton H. T. (2002). Environ. Sci. Technol..

[cit25] Stafiej A., Pyrzynska K., Regan F. (2007). J. Sep. Sci..

[cit26] Ghasemi-Mobarakeh L., Prabhakaran M. P., Morshed M., Nasr-Esfahani M. H., Baharvand H., Kiani S., Al-Deyab S. S., Ramakrishna S. (2011). J. Tissue Eng. Regen. Med..

[cit27] Yang G., Kampstra K. L., Abidian M. R. (2014). Adv. Mater..

[cit28] Kumar P., Kamle M., Singh J., Rao D. P. (2008). J. Biotech. Biochem..

[cit29] Han Q., Shen X., Zhu W., Zhu C., Zhou X., Jiang H. (2016). Biosens. Bioelectron..

[cit30] Yuan L., Zhang J., Zhou P., Chen J., Wang R., Wen T., Li Y., Zhou X., Jiang H. (2011). Biosens. Bioelectron..

[cit31] Wen T., Xue C., Li Y., Wang Y., Wang R., Hong J., Zhou X., Jiang H. (2012). J. Electroanal. Chem..

[cit32] Moraes F. C., Rosii B., Donatoni M. C., de Oliveira K. T., Pereira E. C. (2015). Anal. Chim. Acta.

[cit33] Wang Z., Wang P., Tu X., Wu Y., Zhan G., Li C. (2014). Sens. Actuators, B.

[cit34] Hu L. S., Fong C. C., Zou L., Wong W. L., Wong K. Y., Wu R. S. S., Yang M. (2014). Biosens. Bioelectron..

[cit35] Antoniazzi C., Alves de Lima C., Marangoni R., Spinelli A., Guimaraes de Castro E. (2018). J. Solid State Electrochem..

[cit36] Ni X., Xia B., Wang L., Ye J., Du G., Feng H., Zhou X., Zhang T., Wang W. (2017). Anal. Biochem..

[cit37] Janegitz B. C., dos Santos F. A., Faria R. C., Zucolotto V. (2014). Mater. Sci. Eng., C.

