# Correction: Electrochemical biosensor for detection of 17β-estradiol using semi-conducting polymer and horseradish peroxidase

**DOI:** 10.1039/d0ra90078h

**Published:** 2020-07-20

**Authors:** Kamila Spychalska, Dorota Zając, Aleksandra Wiatrowska, Joanna Cabaj

**Affiliations:** Faculty of Chemistry, Wrocław University of Science and Technology Wybrzeże Wyspiańskiego 27 Wrocław Poland joanna.cabaj@pwr.edu.pl

## Abstract

Correction for ‘Electrochemical biosensor for detection of 17β-estradiol using semi-conducting polymer and horseradish peroxidase’ by Kamila Spychalska *et al.*, *RSC Adv.*, 2020, **10**, 9079–9087, DOI: 10.1039/C9RA09902F.

The authors wish to amend the authorship of this article by adding an additional author, Aleksandra Wiatrowska. The full author list is as shown above.

The Royal Society of Chemistry apologises for these errors and any consequent inconvenience to authors and readers.

## Supplementary Material

